# Dysregulated LIF-STAT3 pathway is responsible for impaired embryo implantation in a Streptozotocin-induced diabetic mouse model

**DOI:** 10.1242/bio.011890

**Published:** 2015-05-22

**Authors:** Tong-Song Wang, Fei Gao, Qian-Rong Qi, Fu-Niu Qin, Ru-Juan Zuo, Zi-Long Li, Ji-Long Liu, Zeng-Ming Yang

**Affiliations:** 1 School of Science, Shantou University, Shantou 515063, China; 2 School of Life Science, Xiamen University, Xiamen 361005, China; 3 Colleage of Veterinary Medicine, South China Agricultural University, Guangzhou 510642, China

**Keywords:** Diabetes, Embryo implantation, Estrogen, LIF-STAT3, Insulin

## Abstract

The prevalence of diabetes is increasing worldwide with the trend of patients being young and creating a significant burden on health systems, including reproductive problems, but the effects of diabetes on embryo implantation are still poorly understood. Our study was to examine effects of diabetes on mouse embryo implantation, providing experimental basis for treating diabetes and its complications. Streptozotocin (STZ) was applied to induce type 1 diabetes from day 2 of pregnancy or pseudopregnancy in mice. Embryo transfer was used to analyze effects of uterine environment on embryo implantation. Our results revealed that the implantation rate is significantly reduced in diabetic mice compared to controls, and the change of uterine environment is the main reason leading to the decreased implantation rate. Compared to control, the levels of LIF and p-STAT3 are significantly decreased in diabetic mice on day 4 of pregnancy, and serum estrogen level is significantly higher. Estrogen stimulates LIF expression under physiological level, but the excessive estrogen inhibits LIF expression. LIF, progesterone or insulin supplement can rescue embryo implantation in diabetic mice. Our data indicated that the dysregulated LIF-STAT3 pathway caused by the high level of estrogen results in the impaired implantation in diabetic mice, which can be rescued by LIF, progesterone or insulin supplement.

## INTRODUCTION

Embryo implantation involves an intricate discourse between the embryo and uterus, including synchronized development of the embryo to the blastocyst stage, differentiation of the uterus to the receptive state, and cross-talk between the blastocyst and uterine luminal epithelium (Lim and Dey, 2009). In rodents, estrogen is essential for preparation of uterine receptivity and embryo implantation. Uterine receptivity is maintained for a limited period. The level of estrogen within a very narrow range determines the duration of the window of uterine receptivity (Ma et al., 2003). A balanced interaction between estrogen and progesterone is required for the establishment of pregnancy (Carson et al., 2000). Perturbation of any of these factors can compromise the establishment of pregnancy and cause infertility (Hewitt and Korach, 2011). Leukemia inhibitory factor (LIF) is highly expressed in mouse uterus during receptivity phase and essential for embryo implantation (Stewart et al., 1992). Signal transducer and activator of transcription 3 (STAT3), a downstream target of LIF, is phosphorylated in the luminal epithelium before embryo implantation (Cheng et al., 2001). The inhibition or uterine conditional deletion of STAT3 leads to the failure of mouse embryo implantation (Nakamura et al., 2006; Sun et al., 2013).

Overweight and obesity are becoming a global problem. Nearly half of all pregnant women are either overweight or obese at conception (Kim et al., 2010). Type 1 diabetes is a common disease in which insulin-producing pancreatic β cells are impaired by immune-mediated mechanisms (Hober and Sauter, 2010). Around 5-10% of diabetic patients are type 1 diabetes which typically manifests in children and adolescents (Jayaraman, 2014). Especially in developing countries, diabetes is less often found or reported. The growing prevalence of diabetes will pose a tremendous challenge to the management of this disease (Bhojani et al., 2013; Kanguru et al., 2014).

Under current treatments, pregnant women with either type 1 or type 2 diabetes often suffer from a series of reproductive problems such as miscarriage, neonatal morbidity and mortality, and congenital malformations (Greene, 1999). Maternal diabetes adversely affects oocyte maturation (Cheng et al., 2011), reduces cell number and protein synthetic rate in blastocysts (Beebe and Kaye, 1991; Wyman et al., 2008), and may further result in post-implantation embryo and placenta developmental abnormalities and even metabolic diseases in the offspring (Diamond et al., 1989; Evers et al., 2003). Insulin is the most commonly used drug to treat type 1 diabetic patients. Currently, treatments for women with type 1 diabetes who are seeking pregnancy include dietary modifications and insulin treatment to help stabilize the blood sugar (Gabbe and Graves, 2003). However, whether and how diabetes affects embryo implantation is poorly understood.

Experimental diabetic models can be induced through surgical procedures, chemical induction, or mutated animal strains. In this study, streptozotocin (STZ) was used to induce type 1 diabetes in mice (Beebe and Kaye, 1991). We showed that the high level of estrogen in diabetic mice may disturb LIF-STAT3 pathway and lead to impaired embryo implantation. LIF, progesterone or insulin supplement can rescue the implantation in diabetic mice.

## RESULTS

### Embryo implantation in diabetic mice

**Fig. 1.**
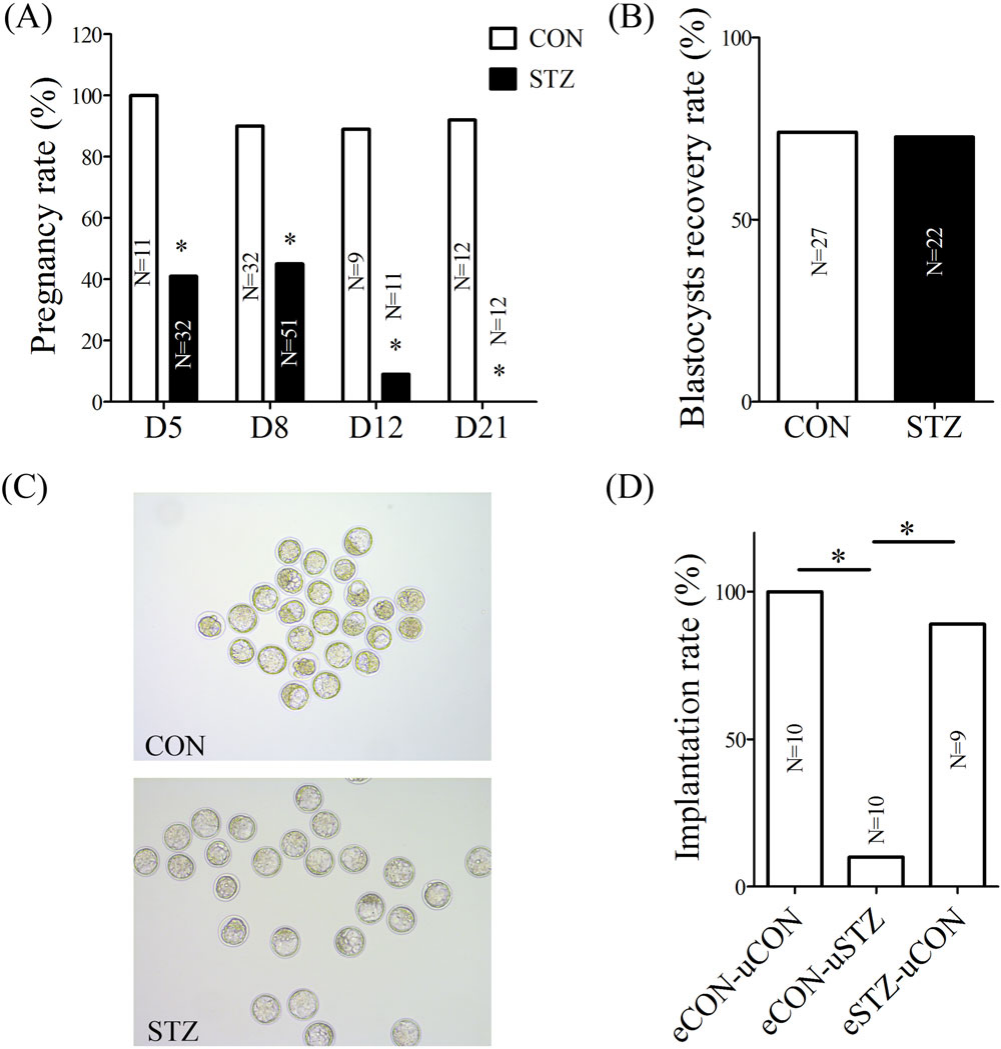
**Embryo implantation rate was reduced in diabetic mice.** (A) Pregnancy rate between control (CON) and diabetic (STZ) mice. (B) Blastocysts recovery rate between control and diabetic mice. (C) Embryos collected from control and diabetic mice. (D) Embryo implantation rate on day 5 of pregnancy after embryos were transferred into day 4 pseudopregnant recipients. eCON-uCON; blastocysts recovered from control mice were transferred into control pseudopregnant recipients. eCON-uSTZ; blastocysts recovered from control mice were transferred into diabetic pseudopregnant recipients. eSTZ-uCON; blastocysts recovered from diabetic mice were transferred into control pseudopregnant recipients. *P<0.05.

In the control group, the pregnancy rate was 100% on day 5, 90% on day 8, 89% on day 12 and 92% on day 21 of pregnancy. However, the pregnancy rate was 41% on day 5, 45% on day 8, 9% on day 12 and 0% on day 21 of pregnancy in the diabetic group (Fig. 1A). The pregnancy rate in the diabetic group was nearly half of that in the control group during early pregnancy.

### The changes of uterine environment in diabetic mice

In order to examine whether the low implantation rate was caused by embryos in the diabetic group, blastocysts were collected and counted from both control and diabetic mice on day 4 of pregnancy. Compared to control, there were no obvious differences in the number and morphology of blastocysts in the diabetic group (Fig. 1B,C). Furthermore, embryo transfer was performed to analyze reasons for the low implantation rate in diabetic group. When blastocysts recovered from control mice were transferred into normal pseudopregnant recipients, the implantation rate was 100%. After blastocysts from diabetic mice were transferred into normal pseudopregnant recipients, the implantation rate was 89%. However, the implantation rate was only 10% when blastocysts from control mice were transferred into diabetic pseudopregnant recipients (Fig. 1D). These results indicated that the change of uterine environment should be the main reason leading to a decreased implantation rate in diabetic mice, rather than those of embryos.

### Abnormal expression of implantation-related genes in diabetic mice

**Fig. 2.**
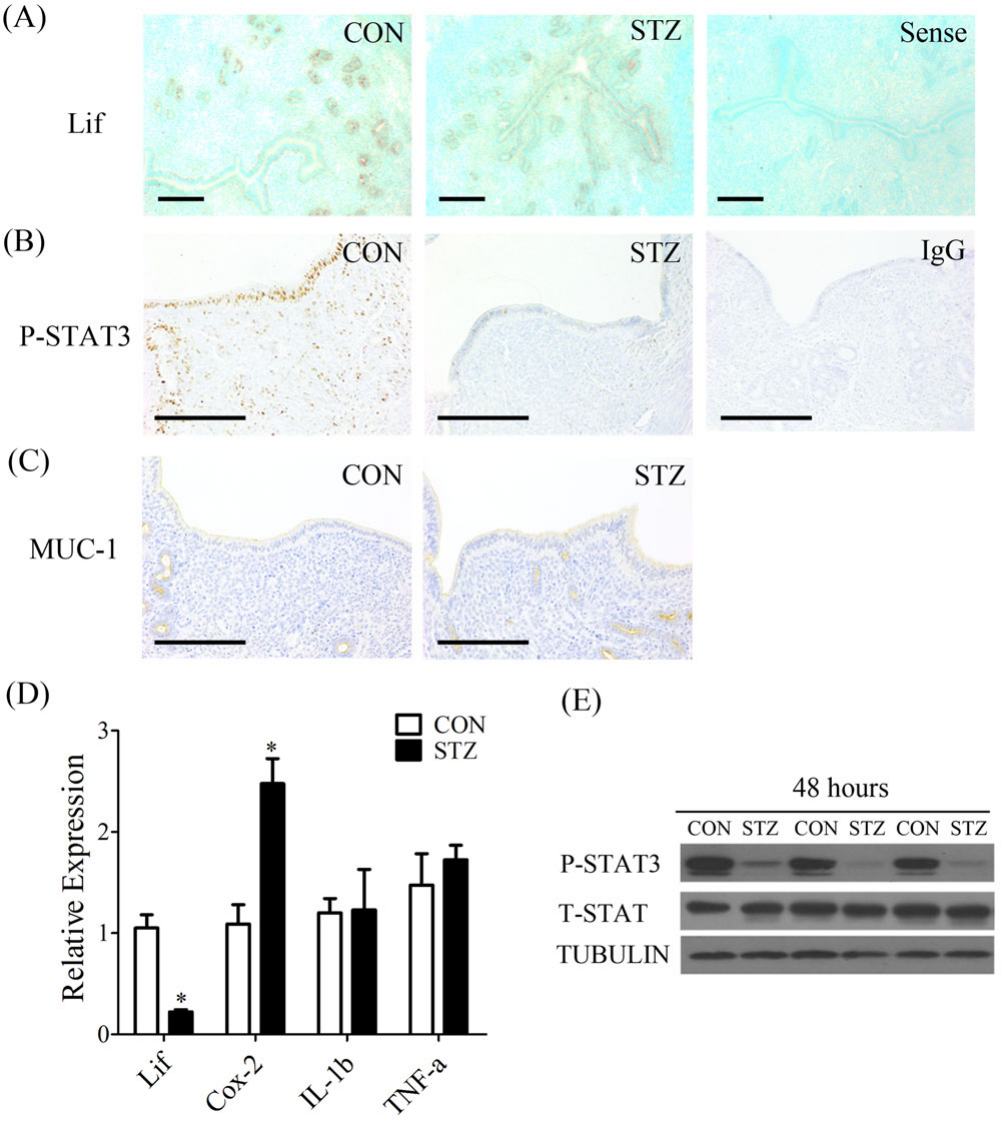
**The change of uterine environment in diabetic mice 48 h after STZ injection.** (A) *In situ* hybridization of *Lif* mRNA in uteri of control (CON) and diabetic (STZ) mice (Sense, negative control). Immunohistochemistry of p-STAT3 (B) and MUC1 (C) in uteri of control and diabetic mice (IgG, negative control). (D) Real-time RT-PCR of *Lif*, Cox-2, IL-1β and Tnfα expression in uteri of control and diabetic mice. (E) Western blot of t-STAT3 and p-STAT3 proteins in uteri of control and diabetic mice. Scale bars, 300 μm. *P<0.05; error bars, s.e.

In order to examine whether the uterine receptivity was changed in diabetic mice, the levels of *Lif* mRNA, and p-STAT3 and Mucin 1 (MUC1) proteins were analyzed by *in situ* hybridization and immunostaining, respectively. *Lif* mRNA expression was primarily detected in the glandular epithelium. Compared to control, *Lif* mRNA signal was lower in diabetic mice (Fig. 2A). The signals of p-STAT3 were mainly observed in luminal epithelium, and weakly in glandular epithelium and stromal cells in control mice. However, p-STAT3 signal was not seen in diabetic mice (Fig. 2B). MUC1 is mainly localized in the luminal epithelium, but the signal is almost undetectable during the implantation window (Li et al., 2011). In our study, immunocytochemical staining analysis confirmed that MUC1 was not expressed in the luminal epithelium in both control and diabetic mice on day 4, while the signal was detected in glandular epithelium in both groups (Fig. 2C).

To investigate the uterine changes after diabetic induction, uteri were collected from diabetic and control mice 48 h after STZ injection. Our results showed that the levels of *Lif* mRNA and p-STAT3 were dramatically reduced at 48 h in diabetic group (Fig. 2D,E). Meanwhile, we analyzed Cyclooxygenase-2 (Cox-2) expression which up-regulated and played an important role in embryo implantation period (Lim et al., 1997). Compared to controls, there was a significant increase of uterine *Cox-2* expression at 48 h (Fig. 2D). Because diabetes can affect the expressions of many cytokines related to immunity and inflammation (Shankar et al., 2011), we examined uterine expressions of tumor necrosis factor α (*TNFα*) and interleukin 1β (*IL-1β*). However, there was no obvious difference between diabetic and control mice (Fig. 2D).

### Dysregulated Lif-STAT3 signal pathway in diabetic uterus

**Fig. 3.**
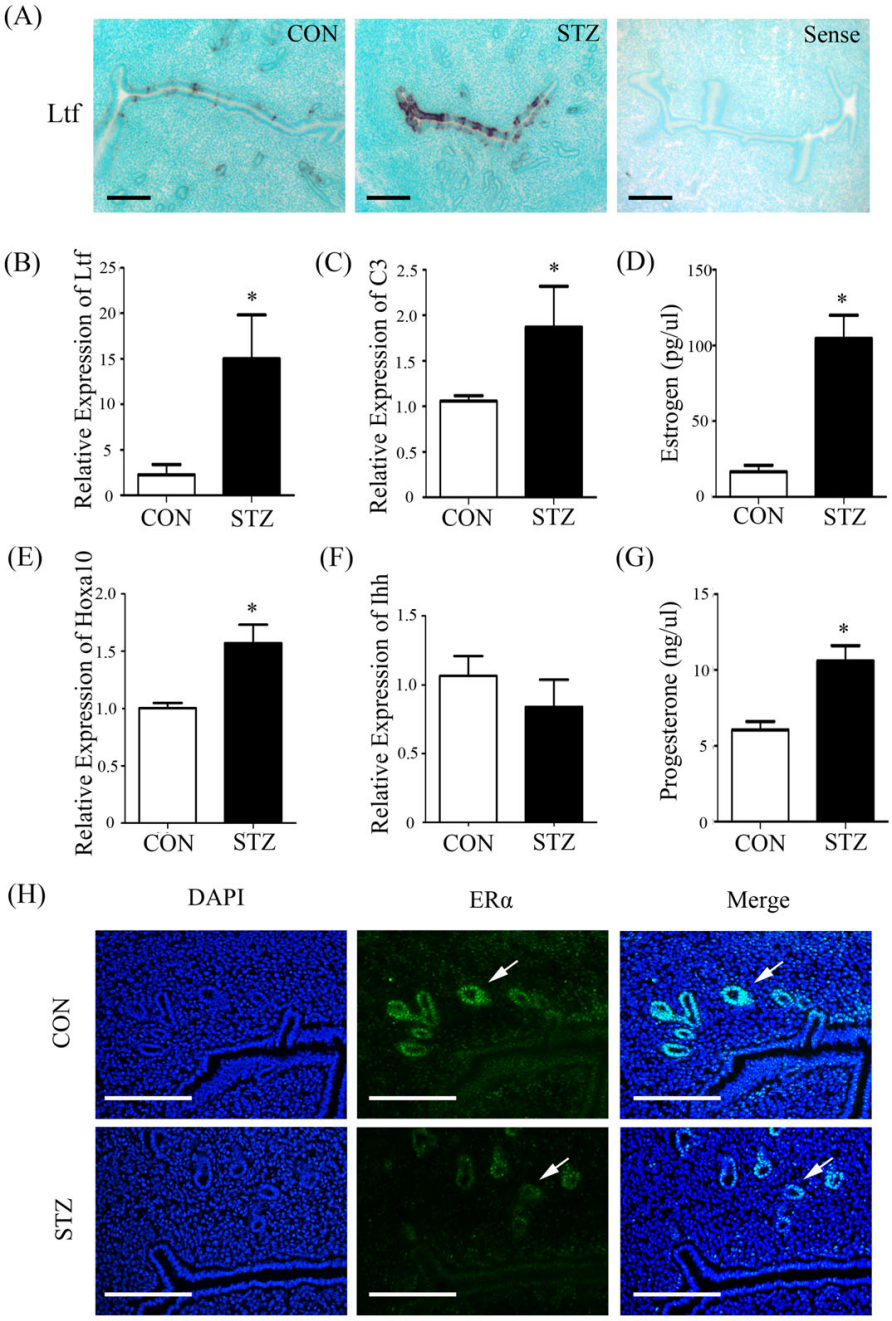
**Estrogen-related changes in control and diabetic mouse uteri on day 4 of pregnancy.** (A) *In situ* hybridization of *Ltf* mRNA in uteri of control (CON) and diabetic (STZ) mice (Sense, negative control). (B) Real-time PCR analysis of *Ltf* expression in uteri of control and diabetic mice. (C) Real-time PCR analysis of *C3* expression in uteri of control and diabetic mice. (D) The serum level of estrogen between control and diabetic mice. (E) Real-time PCR analysis of *Hoxa10* expression in uteri of control and diabetic mice. (F) Real-time PCR analysis of *Ihh* expression in uteri of control and diabetic mice. (G) The serum level of progesterone between control and diabetic mice. (H) Immunofluorescence of ERα expression in uteri of control and diabetic mice (IgG, negative control). Scale bars, 300 μm. *P<0.05; error bars, s.e.

**Fig. 4.**
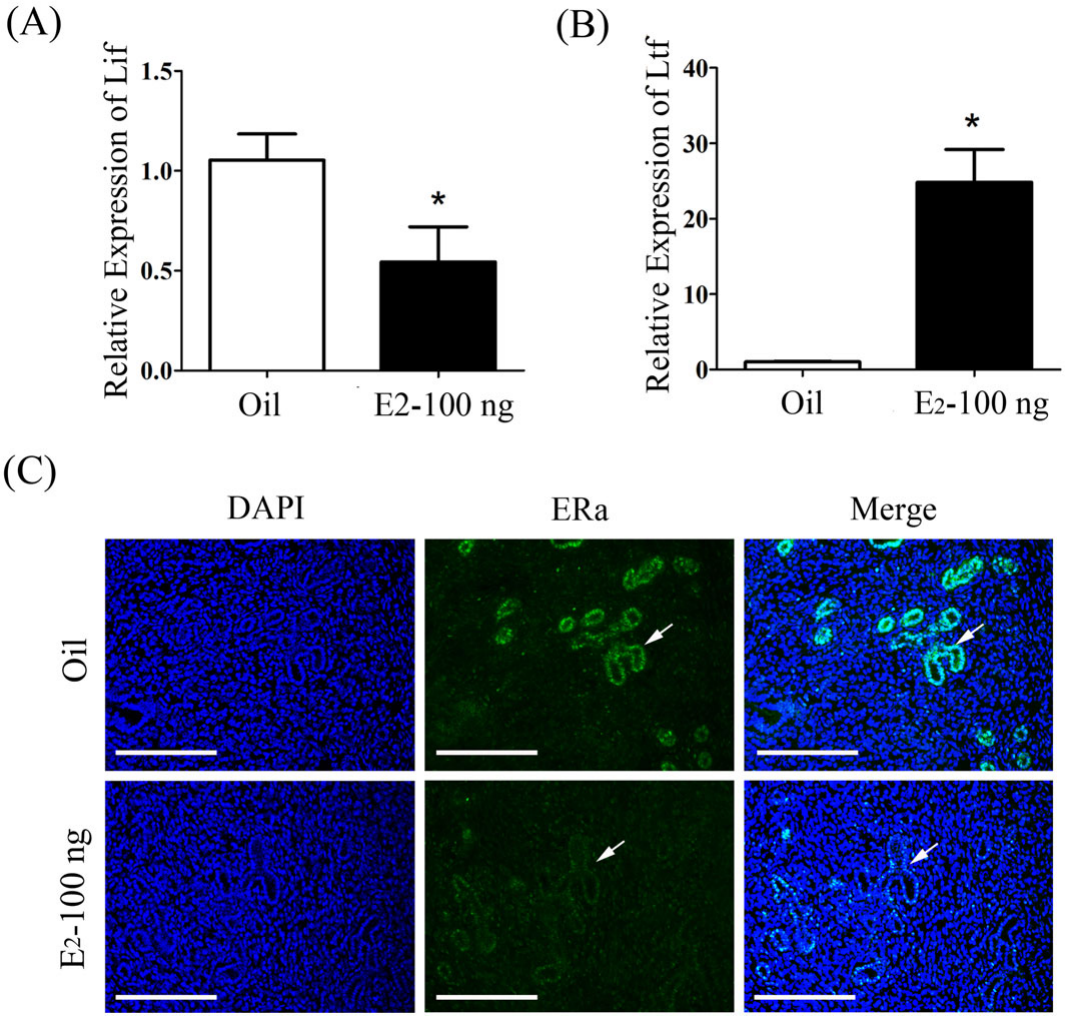
**Effects of estrogen on LIF-STAT3 signal pathway in day 4 pregnant uteri after pregnant mice were treated with estrogen on day 3.** (A) Real-time RT-PCR of *Lif* expression in uteri of pregnant mice treated with oil (Oil) or estrogen (E2-100 ng). (B) Real-time RT-PCR of *Ltf* expression in uteri of pregnant mice treated with oil or estrogen. (C) Immunofluorescence of ERα expression in uteri of pregnant mice treated with oil or estrogen. Scale bars, 300 μm. *P<0.05; error bars, s.e.

Given the special relationship between Lif and estrogen (Bhatt et al., 1991; Yang et al., 1996), we wondered whether estrogen was involved in abnormal changes in LIF-STAT3 signal pathway in diabetic uterus. To confirm this assumption, we examined the expression of lactoferrin (*Ltf*) and complement 3 (*C3*), estrogen-responsive genes, in mouse uterus (McMaster et al., 1992; Sundstrom et al., 1989). Our finding showed that the expression of *Ltf* and *C3* was strikingly up-regulated in diabetic mice than control, which suggested that the level of estrogen may be higher in diabetic mice than control during implantation window (Fig. 3A-C). We also examined the expression of *Hoxa10* (Sroga et al., 2012) and *Ihh* (Simon et al., 2009), progesterone-responsive genes in diabetic mouse uteri. Compared to control, *Hoxa10* expression was slightly increased and *Ihh* expression was not changed in diabetic mice (Fig. 3E,F). Then we found that there were a 6 to 10 fold rise in estrogen level and a 1.5 fold increase in progesterone level in diabetic mice compared with control (Fig. 3D,G), suggesting that diabetes causes a deterioration in hormone homeostasis. Additionally, a significant down-regulation of estrogen receptor alpha (ERα) in glandular epithelium was detected in diabetic female mice on day 4, compared with control (Fig. 3H). In order to analyze whether the down-regulation of *Lif* expression in diabetic mice is caused by excessive estrogen, pregnant mice on day 3 were treated with 100 ng estrogen. Our results showed that *Lif* expression on day 4 was significantly decreased by estrogen treatment (Fig. 4A). In these estrogen-treated mice, *Ltf* expression was obviously increased (Fig. 4B). The expression of ERα was decreased in these estrogen-treated mice (Fig. 4C).

### Rescue of embryo implantation of diabetic mice by LIF or progesterone supplement

**Fig. 5.**
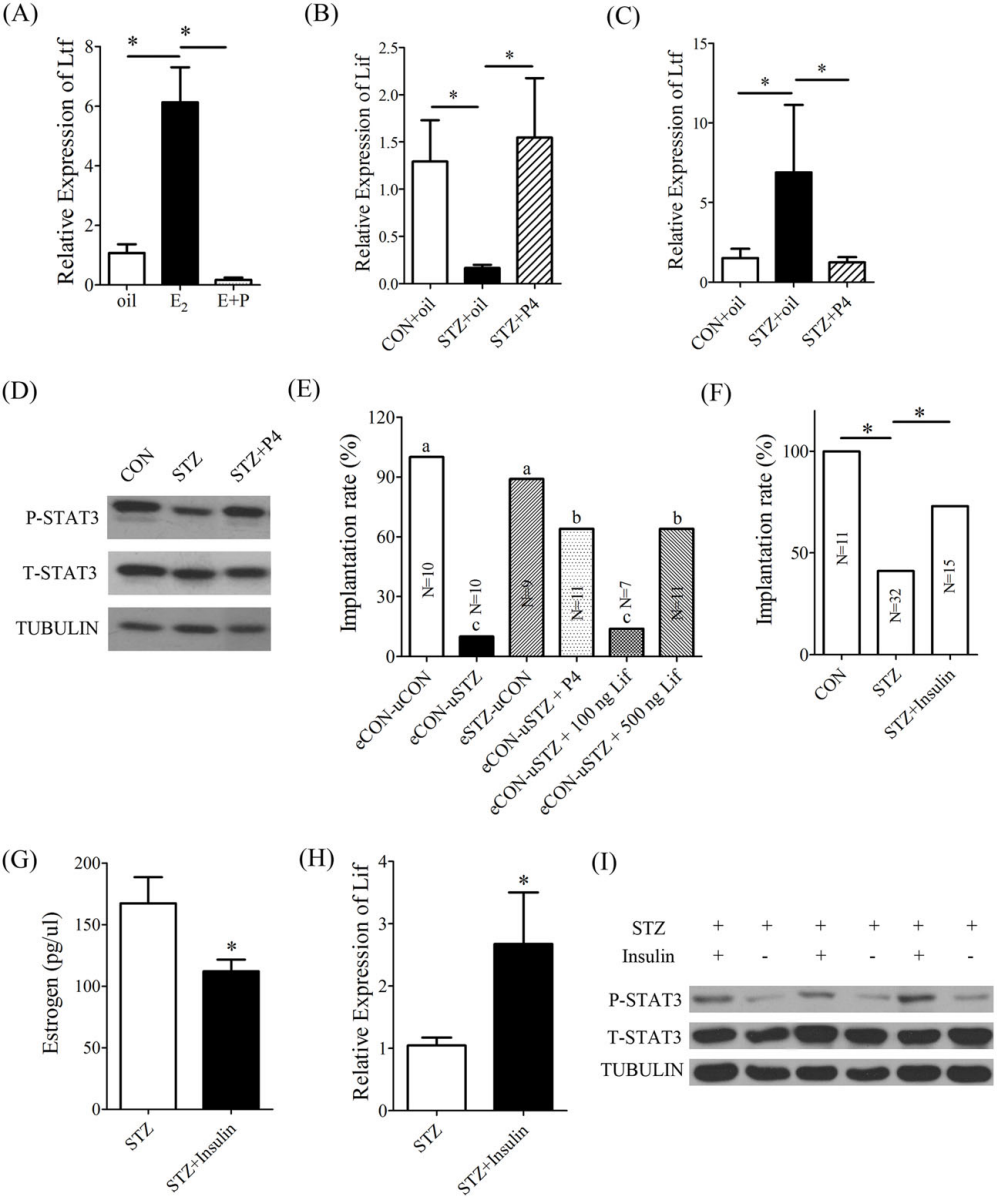
**Effects of LIF, progesterone or insulin supplement on the implantation rate of diabetic mice.** (A) Real-time RT-PCR of *Ltf* expression in the uteri of ovariectomized mice injected with oil, E2, or E2 plus P4 (E+P). (B) Real-time RT-PCR of *Lif* expression in the uteri of control (CON+oil) and diabetic mice injected with oil (STZ+oil) or P4 (STZ+P4). (C) Real-time RT-PCR of *Ltf* expression in the uteri of control and diabetic mice injected with oil or P4. (D) Western blot of t-STAT3 and p-STAT3 proteins in the uteri of control and diabetic mice injected with oil or P4. Tubulin served as a control. (E) Embryo implantation rate on day 5 of pregnancy after embryos were transferred into day 4 pseudopregnant recipients. eCON-uCON, eCON-uSTZ and eSTZ-uCON are described in the legend of Fig. 1. eCON-uSTZ+P4, Blastocysts recovered from control mice were transferred into diabetic pseudopregnant recipients injected with progesterone. eCON-uSTZ+100 ng LIF, Blastocysts recovered from control mice were transferred into diabetic pseudopregnant recipients treated with the intrauterine injection of 100 ng LIF. eCON-uSTZ+500 ng LIF, Blastocysts recovered from control mice were transferred into diabetic pseudopregnant recipients treated with the intrauterine injection of 500 ng LIF. Different lowercase letters in each row show significant differences among different groups (*P*<0.05). (F) Implantation rate of control and diabetic mice treated with insulin. (G) The serum level of estrogen between diabetic mice treated with insulin or not. (H) Real-time RT-PCR of *Lif* expression in uteri between diabetic mice treated with insulin or not. (I) Western blot of t-STAT3 and p-STAT3 proteins in uteri between mice treated with and without insulin. *P<0.05; error bars, s.e.

Based on our data, we assumed that the low implantation rate of diabetic mice was associated with the low *Lif* expression caused by excessive estrogen. Therefore, diabetic mice were supplemented with LIF to examine whether embryo implantation could be rescued. Blastocysts recovered from normal pregnant mice on day 4 were transferred into diabetic pseudopregnant recipients supplemented with the intraluminal injection of 5 µl LIF (100 ng or 500 ng). We found that embryo implantation in diabetic mice was significantly improved by supplementing 500 ng LIF (Figs 5E,7C).

Because progesterone has antagonistic effects on the estrogen-induced proliferation in mouse uterus (Carson et al., 2000), we also checked whether progesterone could counteract estrogen-induced *Ltf* expression in ovariectomized mouse uteri. Compared to estrogen alone, the uterine expression of *Ltf* was significantly down-regulated when ovariectomized mice were treated with both estrogen and progesterone (Fig. 5A). We also examined the expression of *Lif* and *Ltf* in diabetic mice injected with P4 on day 4. Compared to diabetic mice, P4 injection increased the uterine expression of *Lif* and p-STAT3 (Fig. 5B,D), and reduced *Ltf* expression (Fig. 5C). Then progesterone was supplemented into diabetic mice to examine whether embryo implantation could be rescued. Embryo implantation was significantly improved in diabetic mice after blastocysts were transferred into diabetic recipients supplemented with 1 mg progesterone per mouse (Figs 5E,7C). Our results indicated that either Lif or progesterone supplement could rescue embryo implantation in diabetic mice.

### Effects of insulin on embryo implantation in diabetic mice

Insulin is one of the most commonly used treatments for type 1 diabetes. In order to properly carry out complete pregnancy, diabetic women will also continue to use insulin treatment and other means to control blood sugar level during gestation period. Therefore, we used insulin to treat diabetic pregnant mice in our study. The results revealed that insulin treatment significantly improved the implantation rate to 73% (Fig. 5F), down-regulated the estrogen level (Fig. 5G) and increased the levels of Lif (Fig. 5H) and p-Stat3 (Fig. 5I) in diabetic pregnant mice when compared with diabetic mice, which suggested that insulin treatment could partially rescue the dysregulated Lif-STAT3 signal pathway in diabetic uterus.

## DISCUSSION

It is estimated that nearly half of all pregnancies occur in women who are either overweight or obese at conception (Kim et al., 2010). The risk of preeclampsia, gestational diabetes and other labor-related complications are significantly increased in pre-pregnant obese women (King, 2006). Type 1 diabetes typically occurs in children and adolescents with a certain genetic background (Jayaraman, 2014). Type 1 or 2 diabetes is observed in about 3.6% of pregnancies (Al-Rubeaan et al., 2014). The management of diabetes in low- and middle-income countries faces greater challenges compared with those high-income countries (Bhojani et al., 2013; Kanguru et al., 2014). However, whether and how diabetes affects embryo implantation is poorly understood.

In this study, we showed that implantation rate is around 40% in diabetic group compared to control. Compared to normal rats, diabetic rats have a 20% lower implantation rate (De Hertogh et al., 1989). In streptozotocin-induced diabetic pseudopregnant rats, there is a concomitant drop in endometrial decidual growth compared to controls (Zakaria et al., 2000). Our results also showed that insulin treatment could partially reverse the deleterious effects of diabetes on implantation rate.

Previous studies indicated a delay in early embryo development in both chemical-induced and genetically diabetic models (Beebe and Kaye, 1991; Moley et al., 1991). Additionally, there are hyperglycemia-induced metabolic abnormalities in preimplantation embryos in these streptozotocin-induced diabetic mice and some transgenic mice (Moley et al., 1998). Because our diabetic mice were induced on day 2 of pregnancy, we didn't detect any morphologic differences among the blastocysts from diabetic and control mice. Our data from embryo transfer also indicated that blastocysts from diabetic mice can implant in the normal recipients, while blastocysts from control mice have a lower implantation rate in diabetic recipients. These data should exclude the effects of blastocysts from diabetic mice on embryo implantation, suggesting that the low implantation rate in these diabetic mice should be derived from maternal uterus.

During pre-implantation period, the uterus is tremendously sensitive to estrogen level. The duration of the window of uterine receptivity is determined by estrogen within a very narrow range of concentrations and will rapidly become refractoriness at high estrogen levels (Ma et al., 2003). When we examined the levels of endogenous estrogen and progesterone in these diabetic mice, the level of estrogen is more than 6 fold higher than control mice, and progesterone level is also higher than control, but less than 2 folds. Both *Ltf* and *C3*, estrogen-responsive genes, are also significantly upregulated in these diabetic uteri compared to controls. Our data indicated that the super-physiological level of estrogen may compromise embryo implantation in these diabetic mice.

LIF is highly expressed in glandular epithelium on day 4 of pregnancy and stimulated by estrogen (Bhatt et al., 1991; Yang et al., 1996). Embryo implantation fails in LIF-deficient mice (Stewart et al., 1992). LIF can phosphorylate STAT3 via LIF receptor and gp130 (Zhang et al., 1995). Embryo implantation can also be impaired by conditional knockout or inhibition of Stat3 (Lee et al., 2013; Nakamura et al., 2006; Pawar et al., 2013; Sun et al., 2013). In our study, uterine *Lif* expression in diabetic mice is significantly lower than that in control mice. In diabetic mice on day 4 of pregnancy, Stat3 phosphorylation in luminal epithelium is also at a lower level. These data suggested the dysregulated LIF-STAT3 pathway may lead to implantation failure in diabetic mice. A recent study also reported that the levels of LIF and phosphorylated STAT3 are lower in diabetic mice on day 4.5 post-coitum compared to controls (Albaghdadi and Kan, 2012). The rescue of implantation in diabetic mice by LIF supplement also supports this conclusion. It is shown that a high level of estrogen decreases uterine receptivity (Kawagoe et al., 2012; Simon et al., 1995), and the high steroid environment reduces the expression of estrogen receptor alpha (ERα) transcript, because of enhanced receptor processing and degradation (Chai et al., 2011; Wang et al., 2005). In our study, a transient increase of estrogen and significant down-regulation of ERα in glandular epithelium were noticed in diabetic female mice on day 4. The same phenomenon was observed when ovariectomized mice were treated with estrogen. Our results showed that the excessive estrogen inhibits the expression of *Lif* through ERα.

The proliferation and differentiation of endometrial cells are controlled and coordinated by the balanced interaction between estrogen and progesterone. The estrogen-induced proliferation is counteracted by progesterone to ensure the successful establishment of pregnancy (Carson et al., 2000). In PR-Cre-Stat3 deleted mice, estrogen responsive genes are upregulated in uterine epithelial cells (Sun et al., 2013). These studies indicated that a proper balance between estrogen and progesterone is essential to embryo implantation. In this study, we also showed that *Lif* expression is down-regulated when pregnant mice are treated with a high level of estrogen. In ovariectomized mice, estrogen-induced uterine *Lif* mRNA expression is also inhibited when these mice were treated by progesterone. We then found that impaired implantation can be rescued after these diabetic recipient mice are supplemented with progesterone.

In conclusion, our data indicated that the dysregulated LIF-STAT3 pathway caused by high level of estrogen results in the impaired implantation in diabetic mice, which can be rescued by LIF, progesterone or insulin supplement. It is necessary to further determine the levels of LIF and estrogen, and the effects of LIF on implantation defect in diabetic women. If LIF level is low in these women, it is possible to rescue their implantation through LIF or progesterone supplement while they are in the course of insulin or metformin treatment. Our study might help with treating implantation defects in people suffering from diabetes.

## MATERIALS AND METHODS

### Animals and treatments

CD-1 mice were maintained in a controlled environment (12 h light and 12 h dark). All animal procedures were approved by the Institutional Animal Care and Use Committee of Shantou University. To induce pregnancy or pseudopregnancy, adult female mice were mated with fertile or vasectomized males of the same strain (day 1=day of vaginal plug). The implantation sites on day 5 were visualized through intravenous injection of 0.1 ml of 1% Chicago blue dye (Sigma, MO USA) in saline. Ovariectomized mice were treated with different concentration of estradiol-17β (100 ng, 1 μg, or 10 μg/mouse, *s.c*.; Sigma) for 24 h at least 14 days after ovariectomy. Uteri from these mice were collected and frozen into liquid nitrogen for further analysis.

### Generation of diabetic mice and treatments

**Fig. 6.**
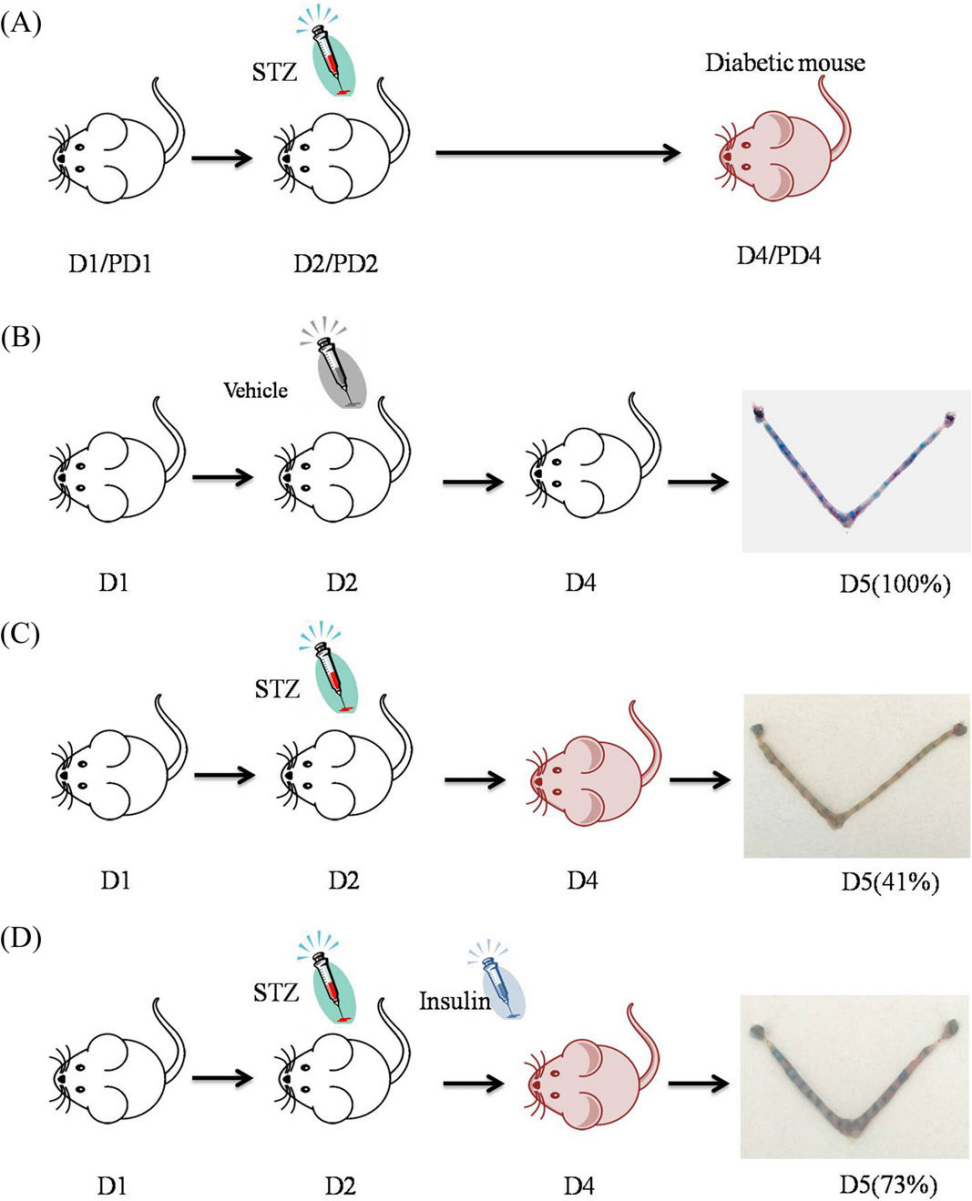
**Generation of diabetic mice and treatments.** (A) Mice on day 2 of pregnancy or pseudopregnancy received a single injection of streptozotocin to induce diabetes. (B) Control mice received an equal volume of the sodium citrate vehicle buffer. The implantation rate on days 5 was 100%. (C) The implantation rate of diabetic mice on days 5 was 41%. (D) The implantation rate on days 5 was 73% while diabetic mice were treated with insulin. The implantation sites on day 5 were visualized through intravenous injection of 0.1 ml of 1% Chicago blue dye in saline. The blue points in uteri in the final image of each panel represent the implantation sites.

To induce diabetes, mice at 9:00 on day 2 of pregnancy or pseudopregnancy received a single injection of streptozotocin (STZ, 190 mg/kg dissolved in 0.1 M sodium citrate buffer, pH 4.4; Sigma) (Fig. 6A,C). Control mice received an equal volume of the sodium citrate vehicle buffer (0.1 M, pH 4.4) (Fig. 6B) (Beebe and Kaye, 1991). Two days after injection on day 4, tail-blood samples were measured for glucose concentrations via a commercial glucometer (Sannuo, Shenzhen, China). Diabetes was defined as blood glucose concentration being ≥16.7 mM. For the insulin treatment group, 2 IU insulin (Sanofi-Aventis, Frankfurt, Germany) was injected *s.c.* into diabetic mice twice at 24:00 on day 3 and 24:00 on day 4 after STZ administration (Fig. 6D). Following insulin injection, glucose level was monitored on days 4 and 5 to ensure that blood glucose returned to control level, respectively. Uteri were collected from diabetic and control mice at 48 and 72 h after STZ injection and frozen into liquid nitrogen for further analysis.

### Embryo transfer

**Fig. 7.**
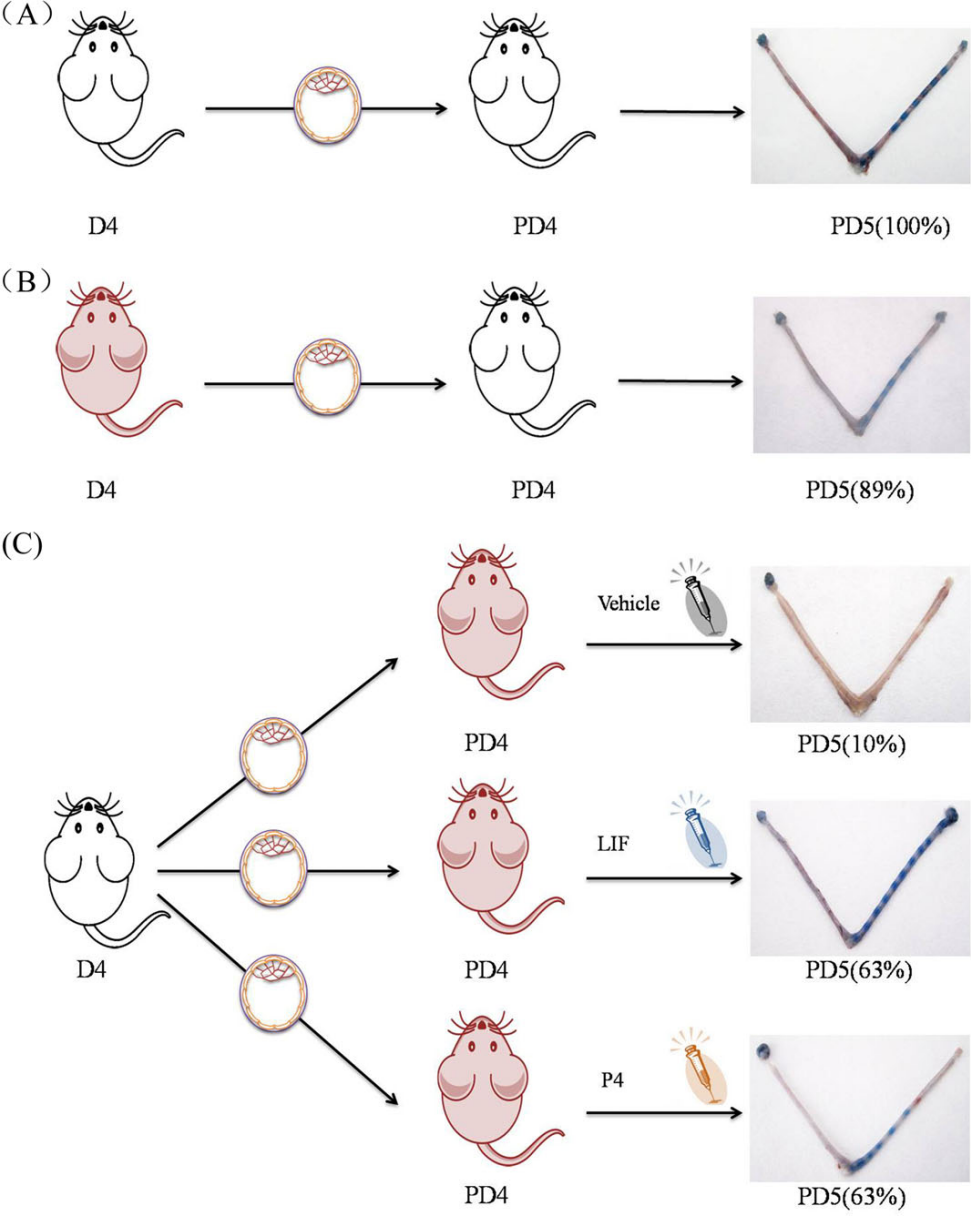
**The model of embryo transplantation and the results.** (A) Blastocysts recovered from control mice were transferred into control pseudopregnant recipients. The implantation rate was 100%. (B) Blastocysts recovered from diabetic mice were transferred into control pseudopregnant recipients. The implantation rate was 89%. (C) Blastocysts recovered from control mice were transferred into diabetic pseudopregnant recipients treated with vehicle, LIF or P4. The implantation rate was 10%, 63% and 63% respectively. The implantation sites on day 5 were visualized through intravenous injection of 0.1 ml of 1% Chicago blue dye in saline. The blue points in uteri in the final image of each panel represent the implantation sites.

Blastocysts were recovered from either STZ-induced diabetic mice or control mice at 9:00 on day 4 of pregnancy, and transferred into nondiabetic pseudopregnant recipients at 10:00 on day 4. There were five groups. Group 1: Blastocysts recovered from control mice were transferred into control pseudopregnant female recipients (Fig. 7A); Group 2: Blastocysts recovered from diabetic mice were transferred into control pseudopregnant female recipients (Fig. 7B); Group 3: Blastocysts recovered from control mice were transferred into diabetic pseudopregnant recipients (Fig. 7C); Group 4: Blastocysts recovered from control mice were transferred into diabetic pseudopregnant female recipients. At the time of embryo transfer, these recipients were treated with the intrauterine injection of LIF (100 ng or 500 ng/mouse) (Fig. 7C); Group 5: Blastocysts recovered from control mice were transferred into diabetic pseudopregnant recipients. At the time of embryo transfer, these recipients were treated with progesterone (*s.c.*, 1 mg/mouse) (Fig. 7C). All mice from the five groups were sacrificed 24 h after transplantation to visualize implantation sites.

### Assay of serum hormones

The orbital blood from each mouse was rested for 30 min at room temperature and spun at a low speed (1000 rpm) for 15 min at 4°C. The supernatants were collected and cryopreserved at −80°C. Serum levels of estrogen and progesterone were determined by estrogen kit (11-Esthu-E01; Alpco, MA USA) and progesterone kit (11-Prohu-E01; Alpco), respectively. Each assay was performed as to the manufacturer's instruction manual.

### Immunohistochemistry

Sections (4 μm thick) of formalin-fixed and paraffin-embedded uteri were deparaffinized, dehydrated and subjected to block with 0.3% hydrogen peroxide. After blocked in 10% horse serum for 1 h at 37°C, sections were incubated with rabbit anti-p-STAT3 (1:200; #9131, Cell Signaling Technology, MA USA) or normal rabbit IgG in 10% horse serum overnight at 4°C. After washing in PBS, the sections were incubated with biotinylated secondary antibody and streptavidin-HRP complex. The positive signals were visualized using DAB Horseradish Peroxidase Color Development Kit according to the manufacturer's protocol (Zhongshan Golden Bridge, Beijing, China) as a reddish-brown color.

### Immunofluorescence

Frozen sections (10 μm thick) were cut with a cryostat, fixed with 4% paraformaldehyde for 1 h and permeabilized with 0.1% Triton X-100 for 20 min. Followed by blocking in 10% horse serum for 1 h at 37°C, sections were incubated with anti-MUC-1 (1:200; NB120-15481, Novus, MO USA) or anti-estrogen receptor α (ERα) antibody (1:400; sc-7207, Santa Cruz, TX USA) overnight at 4°C. After washing in 0.5% Triton X-100/0.5% BSA in PBS, sections were incubated with FITC-conjugated goat anti-rabbit antibody and counter-stained with DAPI for nuclei. Finally, the samples were observed under a fluorescence microscopy.

### Real-time PCR

TRIzol reagent (Invitrogen, CA USA) was used to isolate total RNAs from mouse uteri. After digested with RQ1 deoxyribonuclease I (Promega, WI USA), cDNA was synthesized using Prime Script reverse transcriptase reagent kit (Perfect Real Time; TaKaRa, Dalian, China) and amplified using a SYBR Premix Ex Taq kit (DRR041S; TaKaRa) on the Rotor-Gene 3000A system (Corbett Research, Victoria, Australia). The conditions used for real-time PCR were as follows: 95°C for 10 s, followed by 40 cycles of 95°C for 5 s and 60°C for 34 s. Relative expression levels were calculated using the delta-delta CT (ΔΔCt) method and normalized to Rpl7 expression. The primers used for real-time PCR were: *Lif* (F) AAAAGCTATGTGCGCCTAACA, (R) GTATGCGACCATCCGATACAG; *Cox-2* (F) CCCCCCACAGTCAAAGACACT, (R) GGCACCAGACCAAAGACTTCC; *IL-1β* (F) CTCCATGAGCTTTGTACAAGG, (R) TGCTGATGTACCAGTTGGGG; *TNF-α* (F) ACGTGGAACTGGCAGAAGAG, (R) CTCCTCCACTTGGTGGTTTG; *Ltf* (F) AGCCAACAAATGTGCCTCTTC, (R) CCTCAAATACCGTGCTTCCTC; *C3* (F) TCATCCTCATTGAGACCCCC, (R) CTGCCCCATGTTGACCAGTT; *Rpl7* (F) GCAGATGTACCGCACTGAGATTC, (R) ACCTTTGGGCTTACTCCATTGATA.

### Western blot

Proteins were extracted from uterine tissues with lysis buffer (50 mM Tris-HCl, pH 7.4, 150 mM NaCl, 5 mM EDTA, 10 mM NaF, 1 mM Na3VO3, 1% sodium deoxycholate, 1% Triton X-100 and 0.1% SDS). A complete protease inhibitor (Roche Applied Science, Upper Bavaria, Germany) was added into each sample to prevent protein degradation. Protein concentration was measured with BCA reagent kit (Applygen, Beijing, China). Samples were electrophoretically separated on 10% PAGE and electrically transferred onto PVDF membranes. After blocking with 5% non-fat milk containing 0.5% Tween 20 for 1 h, the membrane was incubated with rabbit anti-total STAT3 antibody (1:1000; #9132, Cell Signaling Technology) or rabbit anti-phosphorylated (Tyr 705) STAT3 antibody (1:1000; #9131, Cell Signaling Technology) overnight at 4°C. The membrane was then incubated with HRP-conjugated secondary antibody (1:5000) for 1 h. Signals were analyzed by ECL Chemiluminescent kit (Amersham Pharmacia Biotech, IL USA). Tubulin was used as a loading control.

### *In situ* hybridization

Digoxigenin-labeled antisense or sense RNA probes were transcribed *in vitro* using a digoxigenin RNA labeling kit (Roche Applied Science). Liquid nitrogen-frozen uteri were cut into 10 μm frozen sections. Frozen sections were mounted on 3-aminopropyltriethoxy-silane (Sigma) coated slides and fixed in 4% paraformaldehyde (Sigma) solution. Hybridization was performed at 55°C for 16 h as previously described (Liang et al., 2014). Then sections were incubated in sheep anti-digoxigenin antibody conjugated to alkaline phosphatase (1:5000; 11207741910, Roche Applied Science). The signal was visualized with the buffer containing 5-bromo-4-chloro-3-indolyl phosphate (0.4 mM) and nitro blue tetrazolium (0.4 mM). Levamisole (2 mM, Sigma) was used to inhibit the endogenous alkaline phosphatase activity. All of the sections were counterstained with 1% methyl green and the positive signal was visualized as dark brown. Sections hybridized with sense probe of each gene were served as negative controls. No detectable signals were observed with sense probes.

### Statistical analysis

At least three replicates were conducted for each treatment. All data were analyzed with ANOVA. A Duncan's multiple comparison test was used to analyze differences. All of the analyses were performed using GraphPad prism software (GraphPad, Inc., CA USA). Data are expressed as mean±s.d. *P*<0.05 was considered significant.
